# Supplementary material for: One-step N-Terminomics Based on Isolation of Protein N-Terminal Peptides From LysargiNase Digests by Tip-Based Strong Cation Exchange Chromatography
Source: Mol Cell Proteomics. 2024 Jul 26;23(9):100820. doi: 10.1016/j.mcpro.2024.100820 (PMC11382313; doi:10.1016/j.mcpro.2024.100820)
Supplement: Supplemental data [file mmc1.pdf]

Supplemental Data

One-step N-terminomics based on isolation of protein N-terminal peptides from LysargiNase digests by tip-based strong cation exchange chromatography

Kazuya Morikawa[1], Hiroshi Nishida[1], Koshi Imami[1,2], Yasushi Ishihama[1,3]

1) Department of Molecular Systems BioAnalysis, Graduate School of Pharmaceutical Sciences, Kyoto University, Kyoto 606–8501, Japan
2) Proteome Homeostasis Research Unit, RIKEN Center for Integrative Medical Sciences, Yokohama 230-0045, Japan
3) Laboratory of Clinical and Analytical Chemistry, National Institute of Biomedical Innovation, Health and Nutrition, Ibaraki, Osaka 567-0085, Japan.

*Correspondence and requests for materials should be addressed to Y.I. (email: yishiham@pharm.kyoto-u.ac.jp).

Table of contents

Figure S1. Schematic illustration of protein N-terminal peptide enrichment.

Figure S2. Insufficient separation of protein N-terminal peptides under formic acid-based isocratic condition. SCX tip-based separation of LysargiNase-digested HEK293T peptides under formic acid-based isocratic condition.

Figure S3. Comparison of salt-based and acid-based isocratic elution of protein N-terminal peptides without fractionation.

Figure S4. Characteristics of the neo-Nt-peptides. Peptides identified in at least one of the three replicates were analyzed.

Figure S5. MS/MS spectra of peptides matched to transit peptide or signal peptide cleavage site.

Figure S6. Characteristics of the peptides identified by each of the three CHAMP methods.

Table S1. N-Terminal peptide elution conditions.

Table S2. Identification list of 2.5% FA, 1 fraction samples.

Table S3. Identification list of 2.5% FA, 6 fractions samples.

Table S4. Identification list of salt-based elution or 0.2% TFA, 6 fractions samples.

Table S5. Identification list of 0.5% TFA, 4 fractions samples.

Table S6. Identification list of salt-based elution or 0.2% TFA or 0.5% TFA, 1 fraction samples.

Table S7. Identification list of 2.5% FA or 0.5% TFA, 1 fraction samples.

Table S8. Identification list of HPLC-based or tip-based isolation samples.

Table S9. Identification list of neo-N-terminal search samples.

Table S10. Identification list of 3 CHAMP methods-based comprehensive protein N- and C-terminomics samples.

Figure S1

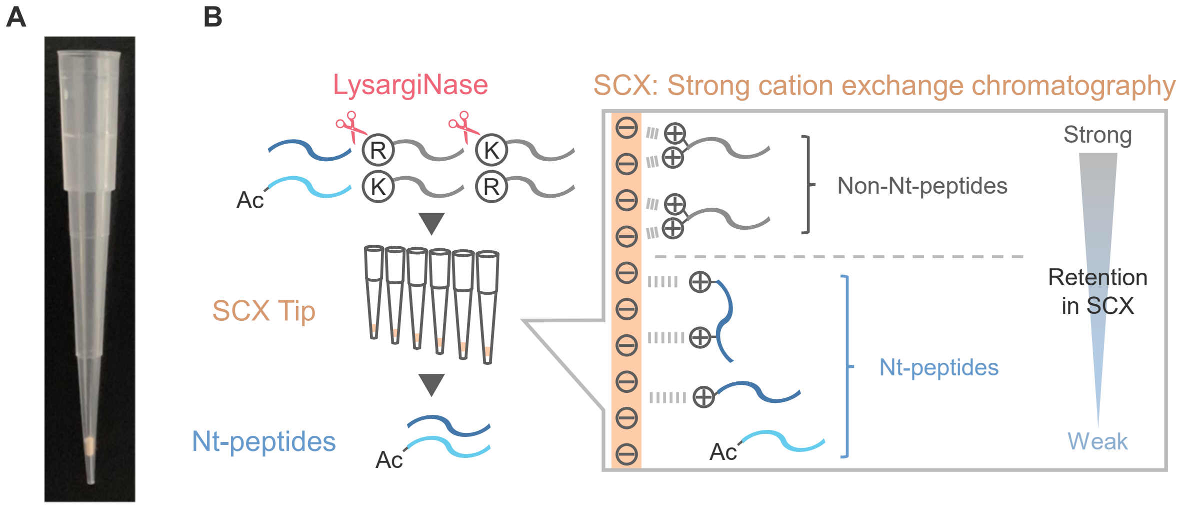

Figure S1. Schematic illustration of protein N-terminal peptide enrichment. (A) Photograph of an SCX-StageTip column. (B) Proteins are digested with LysargiNase, and tip-based SCX chromatography is performed to trap the non-N-terminal peptides. The non-N-terminal peptides are retained in the SCX column, whereas the protein N-terminal peptides flow through the SCX column.

Figure S2

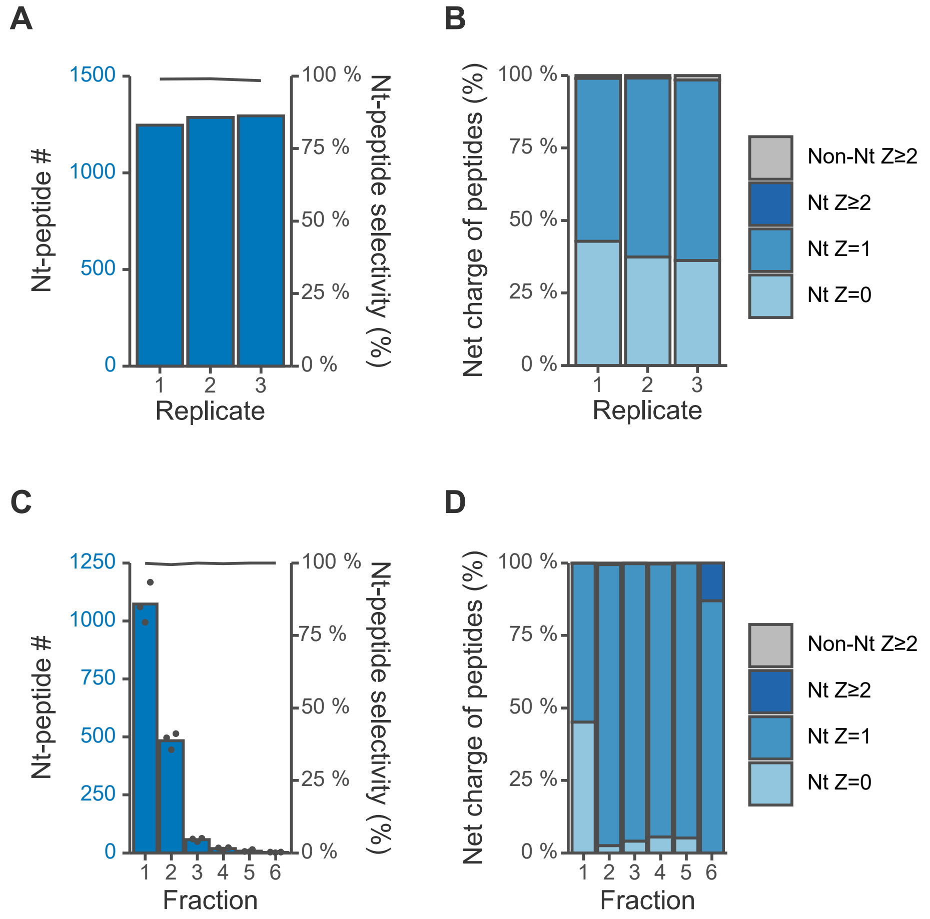

Figure S2. Insufficient separation of protein N-terminal peptides under formic acid-based isocratic condition. SCX tip-based separation of LysargiNase-digested HEK293T peptides under formic acid-based isocratic condition. Z is the charge number at acidic pH, which is based on the number of basic residues per peptide, such as unmodified N-terminus, Lys, Arg and His. The Orbitrap Fusion Lumos system was used. (A) Numbers of identified protein N-terminal peptides. N-Terminal peptide selectivity (%) was calculated as the sum of the signal intensity of all identified N-terminal peptides divided by that of all identified peptides. (B) Distributions of Z values of identified peptides. (C) Numbers of identified protein N-terminal peptides with fractionation.(D) Distributions of Z values of identified peptides with fractionation.

Figure S3

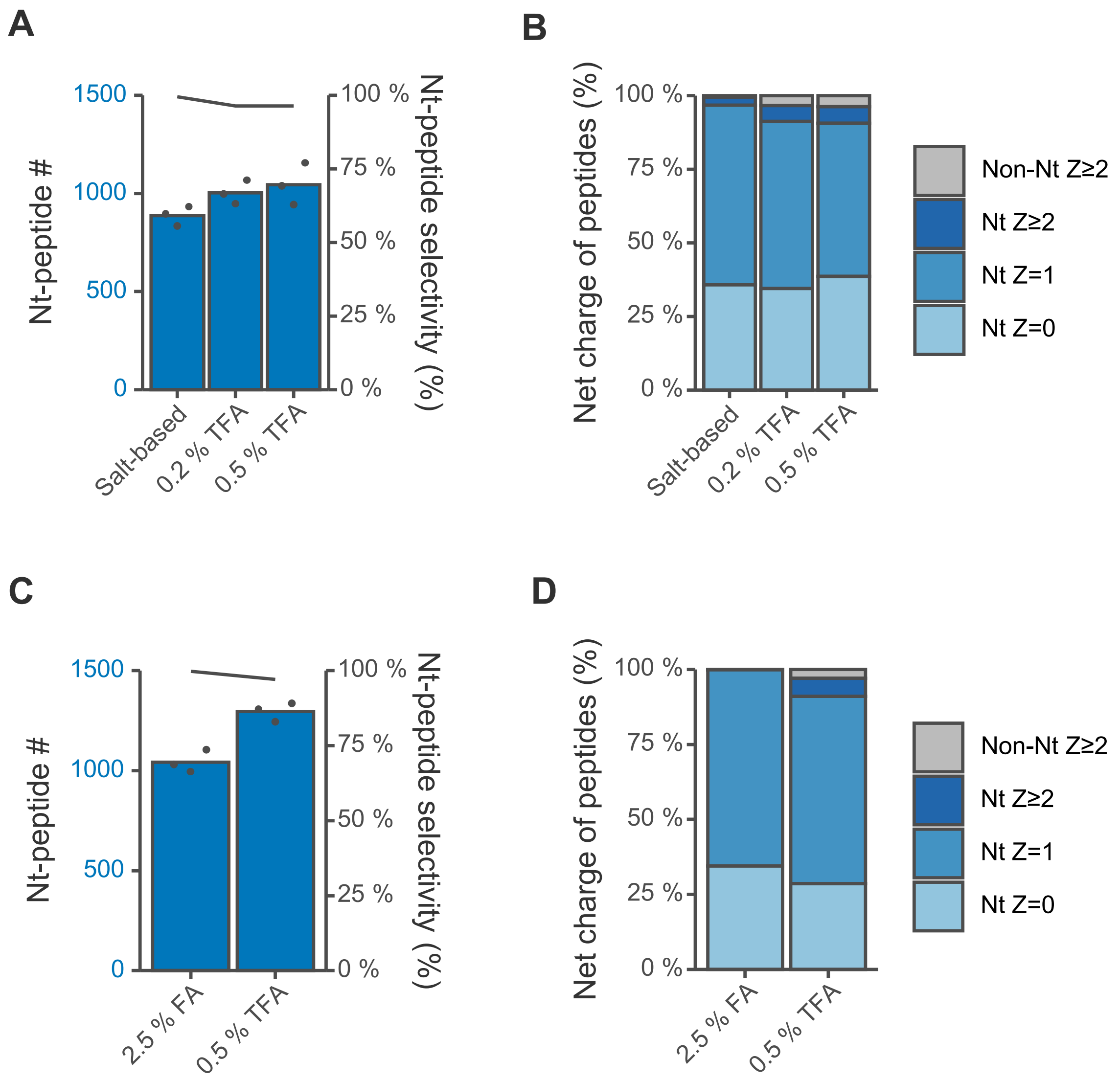

Figure S3. Comparison of salt-based and acid-based isocratic elution of protein N-terminal peptides without fractionation. SCX tip-based separation of LysargiNase-digested HEK293T peptides under isocratic conditions with salt or acid. Z is the charge number at acidic pH, which based on the number of basic residues per peptide, such as unmodified N-terminus, Lys, Arg and His.(A) & (C) Numbers of identified N-terminal peptides. N-Terminal peptide selectivity (%) was calculated as the sum of the signal intensity of all identified N-terminal peptides divided by that of all identified peptides. (B) & (D) Distributions of Z values of identified peptides.

Figure S4

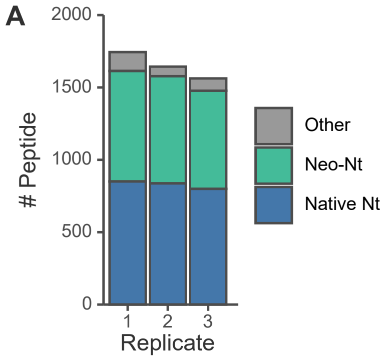

B

UniProt ID	Gene name	Identified sequence	mRNA sequence around the TIS candidate
P10412	H1-4	Ac-AAAGYDVEKNNS	…CUG/GCAGCC…
Q8IWJ2	GCC2	Ac-QETVSKEAEL	…CUG/CAGGAA…

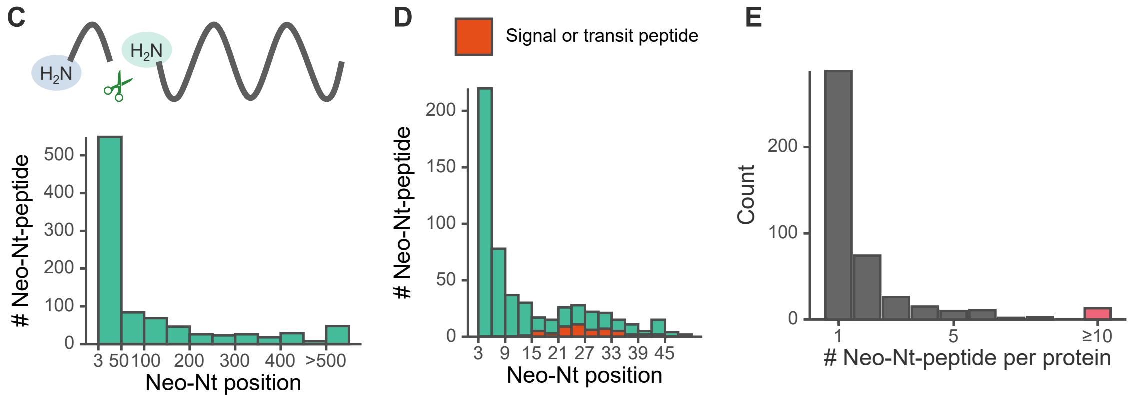

F

UniProt ID	Gene name	Start position
P22626	HNRNPA2B1	1; 24; 26; 29; 70; 133; 191; 245; 246; 247; 248; 301; 326; 328
P62937	PPIA	1; 2; 3; 4; 9; 82; 93; 99; 101; 103; 104; 105; 107; 109; 155
P27816	MAP4	2; 3; 4; 5; 6; 7; 8; 10; 11; 12; 13; 15; 120; 414; 482; 809
P60709	ACTB	2; 6; 8; 9; 10; 17; 50; 52; 218; 219; 222; 238; 239; 240; 241; 300; 315
P06454	PTMA	2; 3; 4; 5; 6; 7; 8; 68; 71; 72; 73; 74; 77; 78; 79; 90; 95
P16403	H1-2	2; 3; 4; 5; 6; 7; 8; 9; 10; 11; 12; 13; 14
P10412	H1-4	2; 3; 4; 5; 6; 7; 8; 9; 11; 12; 13; 14; 34; 36; 67
P16402	H1-3	2; 3; 4; 5; 6; 8; 9; 11
P11142	HSPA8	2; 36; 142; 147; 159; 288; 428; 430; 431; 432; 433; 434; 436; 437; 622
Q13263	TRIM28	6; 7; 8; 9; 10; 11; 13; 14; 20; 102; 444; 493; 510; 676
P16104	H2AX	57; 58; 59; 60; 62; 101; 104; 105; 106; 107; 108; 135
P06748	NPM1	88; 91; 170; 175; 176; 177; 178; 180; 181; 182; 184; 279
P10809	HSPD1	96; 145; 146; 238; 271; 272; 273; 274; 275; 277; 376; 405; 430; 433; 434; 448; 449; 481

Figure S4. Characteristics of the neo-Nt-peptides. Peptides identified in at least one of the three replicates were analyzed. The Orbitrap Exploris 480 system was used. (A) Numbers of identified peptides with semi-specific search free N-terminus. (B) translation initiation site candidates with near-cognate codons. (C) Neo-Nt starting position of identified neo-Nt-peptides. (D) Zoom into the 3-50 neo-Nt starting position. Transit peptide or signal peptide cleavage site matched peptides are shown in red. (E) Numbers of neo-Nt-peptides per protein. (F) List of proteins with ≥10 neo-Nt-peptides. Peptide ladder sequences (peptides with three or more consecutive start positions) are shown in red.

Figure S5

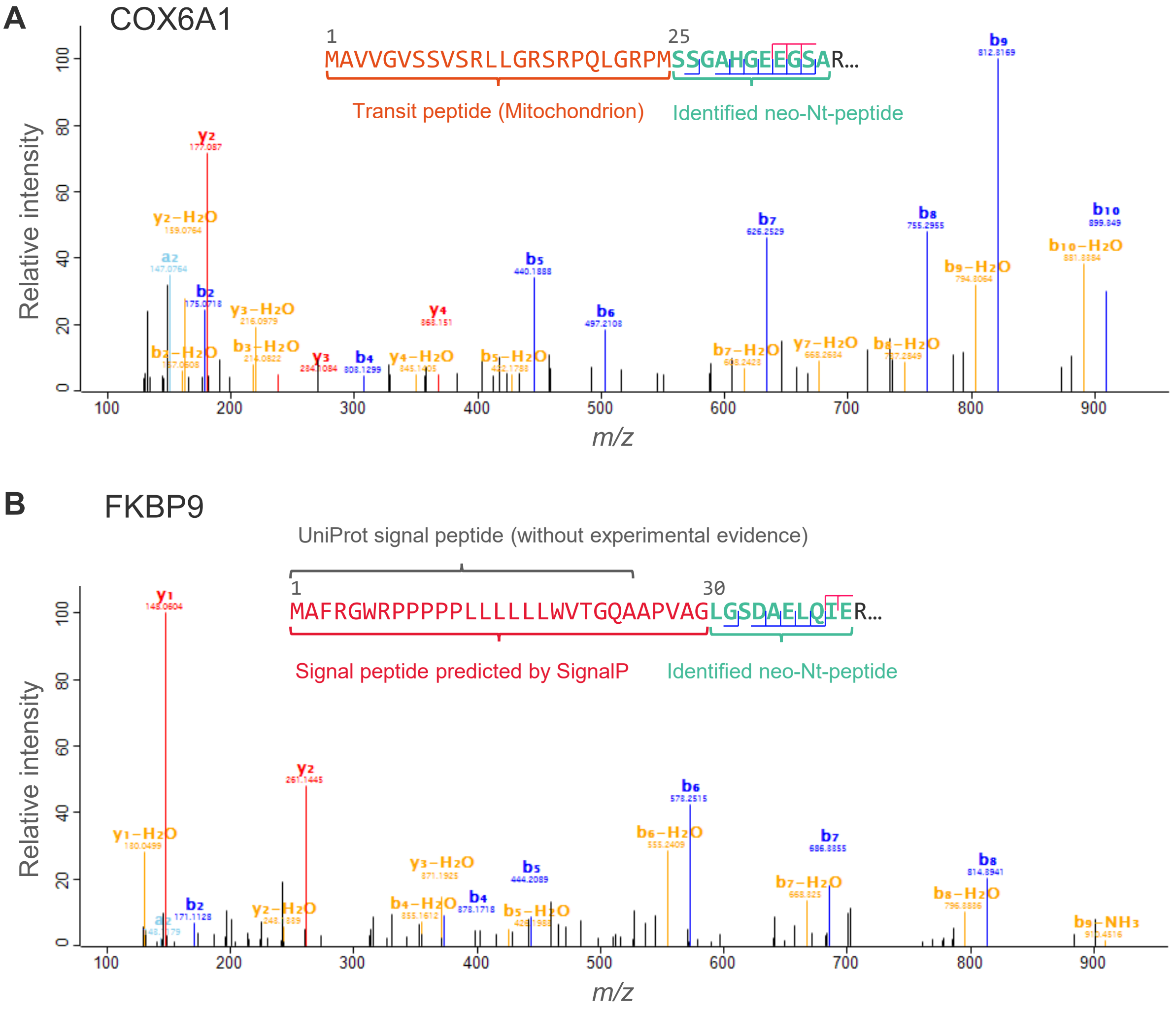

Figure S5. MS/MS spectra of peptides matched to transit peptide or signal peptide cleavage site. The Orbitrap Exploris 480 system was used. (A) MS/MS spectrum of the peptide matched to the transit peptide cleavage site annotated in UniProt. (B) MS/MS spectrum of the peptide matched to the signal peptide cleavage site predicted by SignalP 6.0.

Figure S6

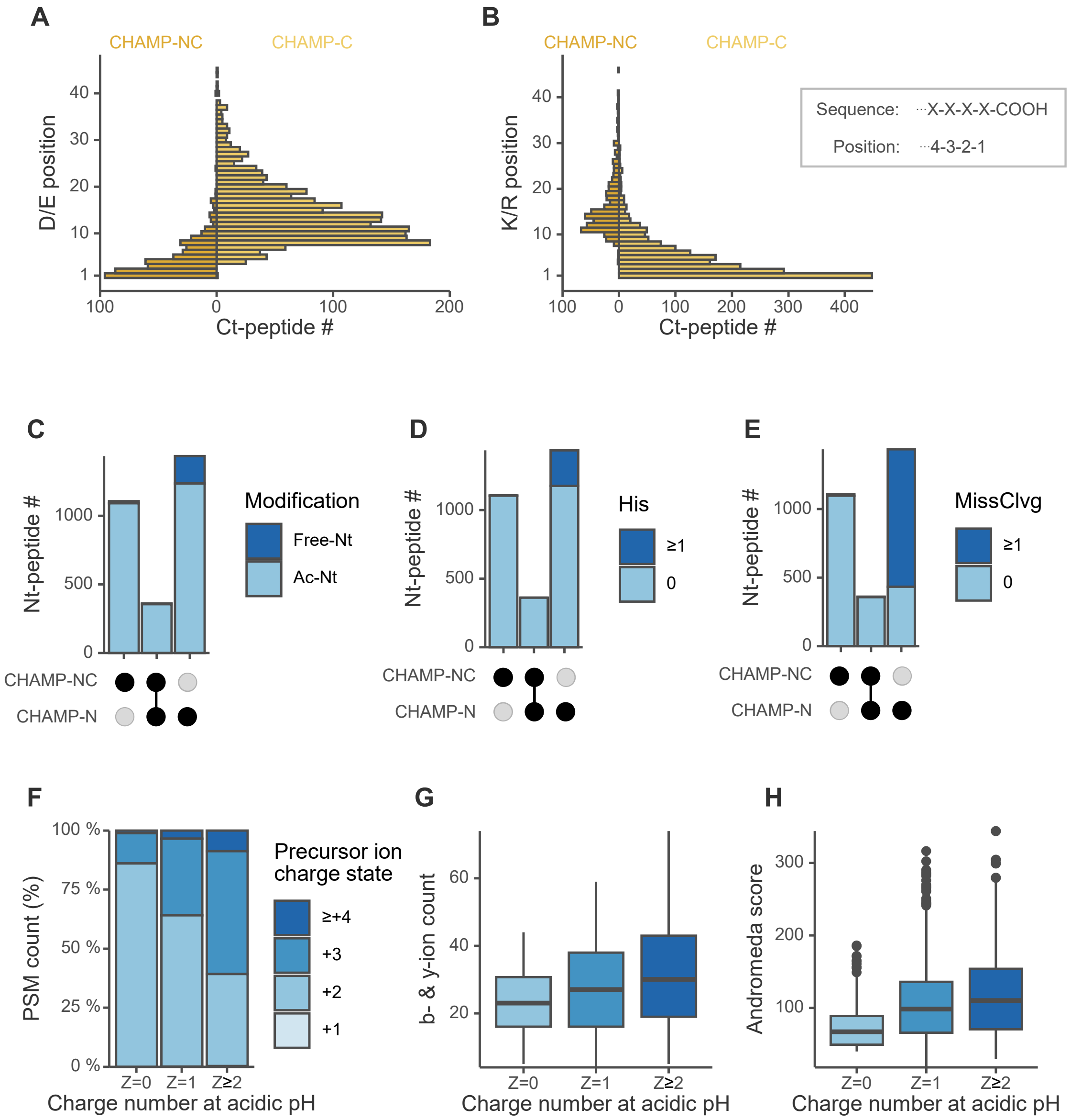

Figure S6. Characteristics of the peptides identified by each of the three CHAMP methods.

Peptides identified in at least one of the three replicates were analyzed. The Orbitrap Exploris 480 system was used. (A) The residue distance distribution of the nearest Asp or Glu residue to the identified peptide sequence (including residues adjacent to the V8 protease digestion site) in CHAMP-NC and CHAMP-C experiments. (B) The residue distance distribution of the nearest Lys or Arg residue to the identified peptide sequence (including residues adjacent to the trypsin digestion site) in CHAMP-NC and CHAMP-C experiments. (C) Acetylation states of N-terminal peptides identified by CHAMP-N or CHAMP-NC. (D) Numbers of His residues in the N-terminal peptides identified by CHAMP-N or CHAMP-NC. (E) Number of missed cleavages in the N-terminal peptides identified by CHAMP-N or CHAMP-NC. For peptides identified by both CHAMP-N and CHAMP-NC, missed cleavages in trypsin digested peptides were counted. (F) Distributions of precursor ion charge state of Nt-peptides identified by CHAMP-N. (G) Relationship between charge number at acidic pH and b- and y-ion count of Nt-peptides identified by CHAMP-N. (H) Relationship between charge number at acidic pH and Andromeda score (MaxQuant) of Nt-peptides identified by CHAMP-N.
